# Supplementary material for: Serra da Estrela PDO Cheese Microbiome as Revealed by Next Generation Sequencing
Source: Microorganisms. 2021 Sep 22;9(10):2007. doi: 10.3390/microorganisms9102007 (PMC8537266; doi:10.3390/microorganisms9102007)
Supplement: Supplementary file 1 [file microorganisms-09-02007-s001.zip › Supplementary material file S1.pdf]

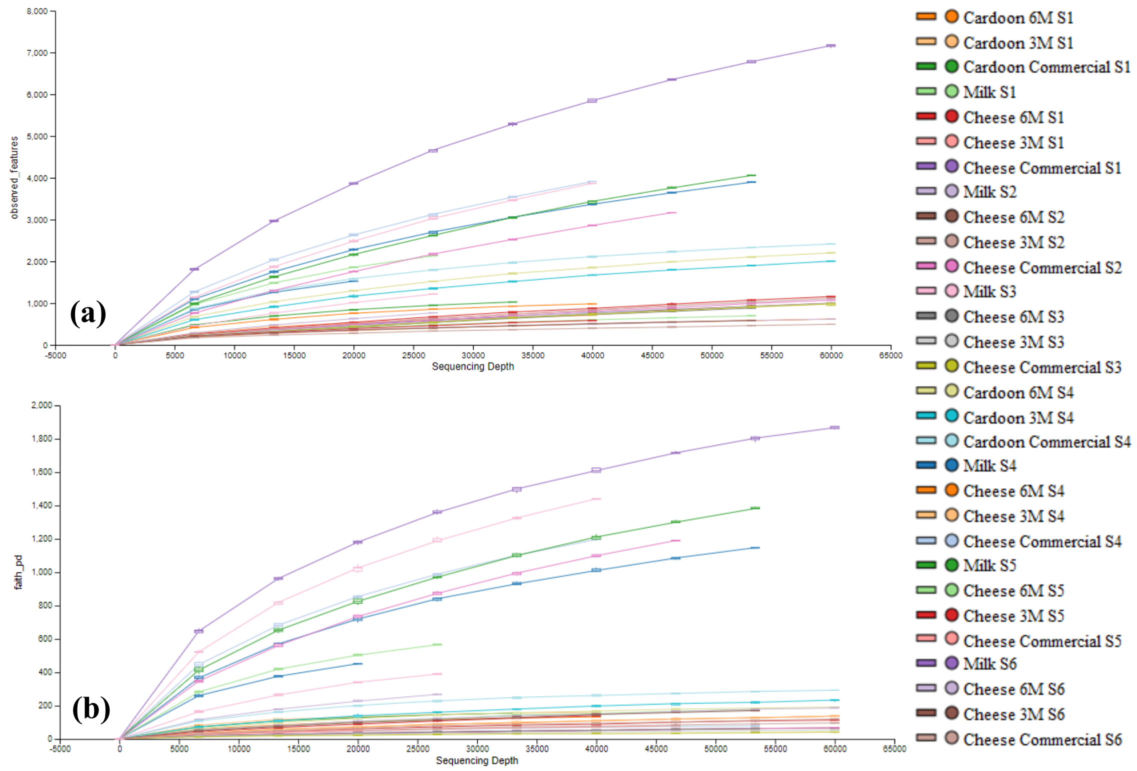

Figure S1 – Rarefaction curves indicative of (a) observed OTUs and (b) Faith diversity obtained from V3−V4 region sequencing of each raw ewes' milk, cardoon and Serra da Estrela cheese DNA samples analysed over two production campaigns, 2018/2019 (S1 to S3) and 2019/2020 (S4 to S6) and at three different time points within each campaign, November – January (S1 and S4), February – March (S2 and S5) and May – June (S3 and S6).

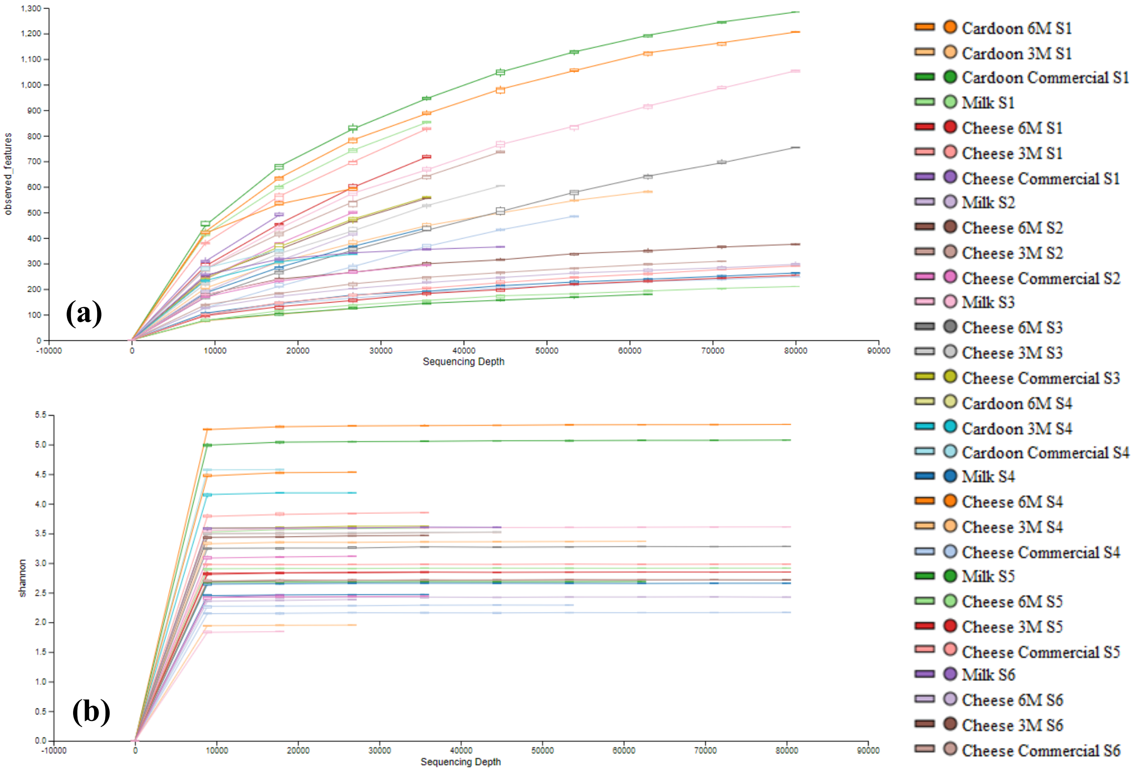

Figure S2 – Rarefaction curves indicative of (a) observed OTUs and (b) Shannon diversity obtained from Internal Transcribed Spacer 2 region sequencing of each raw ewes' milk, cardoon and Serra da Estrela cheese DNA samples analysed over two production campaigns, 2018/2019 (S1 to S3) and 2019/2020 (S4 to S6) and at three different time points within each campaign, November – January (S1 and S4), February – March (S2 and S5) and May – June (S3 and S6).

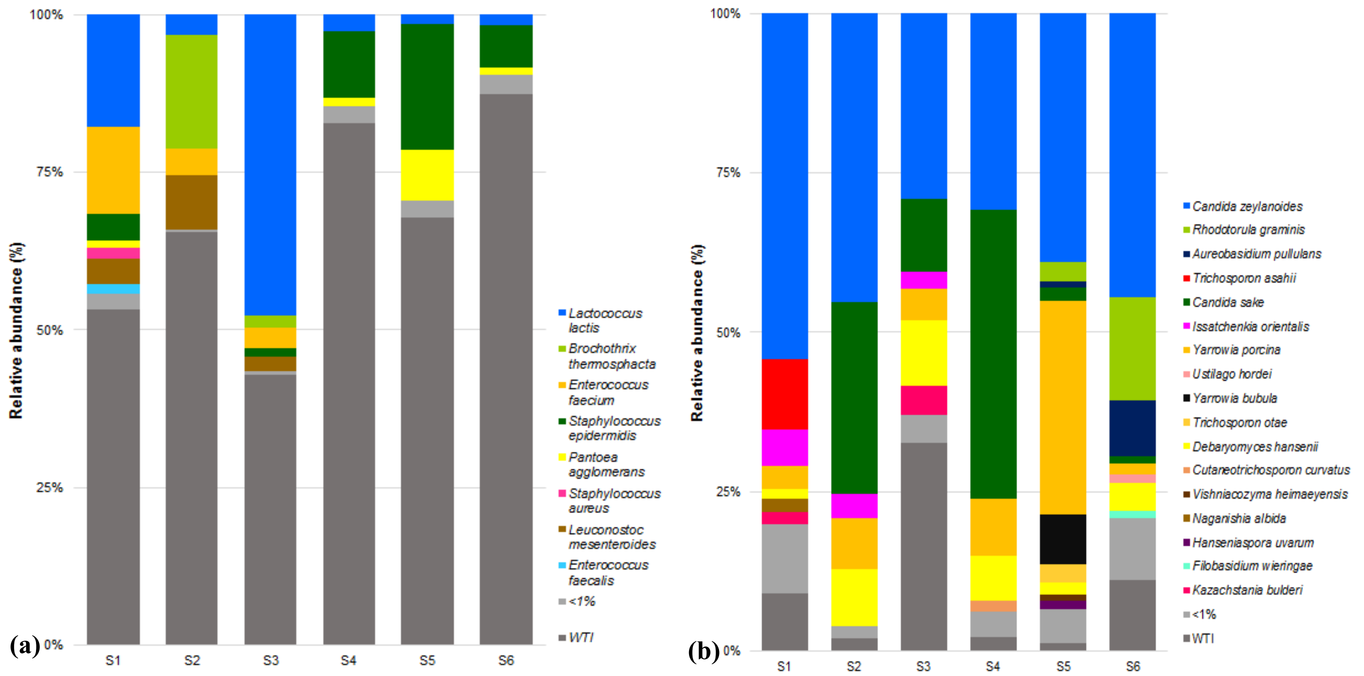

Figure S3 – Relative abundances (%) of dominant sequences (>1%) assigned to species level identified in raw ewes' milk DNA samples based on partial sequence analysis of the (a) V3−V4 and (b) Internal Transcribed Spacer 2 regions of the rRNA gene. WTI refers to percentage of sequences without taxonomic attribution to the specified taxonomic level.

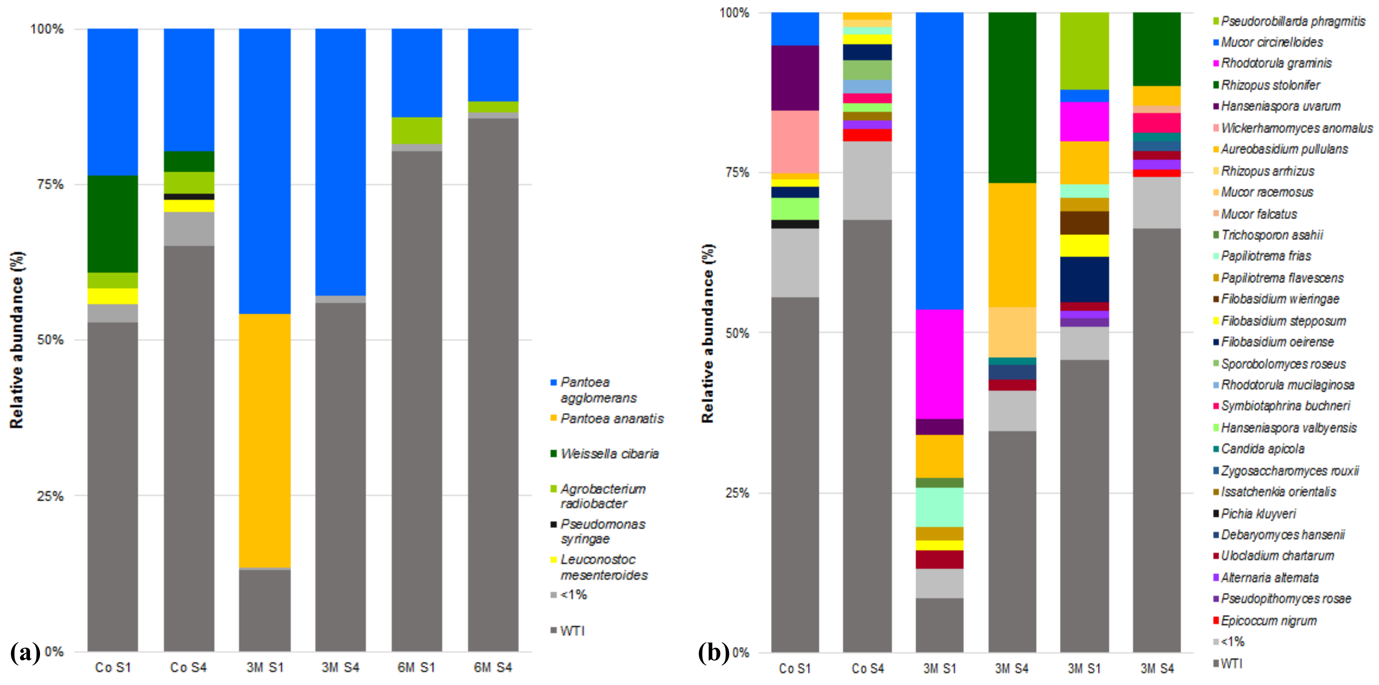

Figure S4 – Relative abundances (%) of dominant sequences (>1%) assigned to species level identified in dried flowers of *Cynara cardunculus* L. (cardoon) DNA samples based on partial sequence analysis of the (a) V3−V4 and (b) Internal Transcribed Spacer 2 regions of the rRNA gene. Co refers to the commercially used cardoon by the cheese producer and 3M and 6M refers to specific cardoon genotypes. WTI refers to the percentage of sequences without taxonomic attribution to the specified taxonomic level.

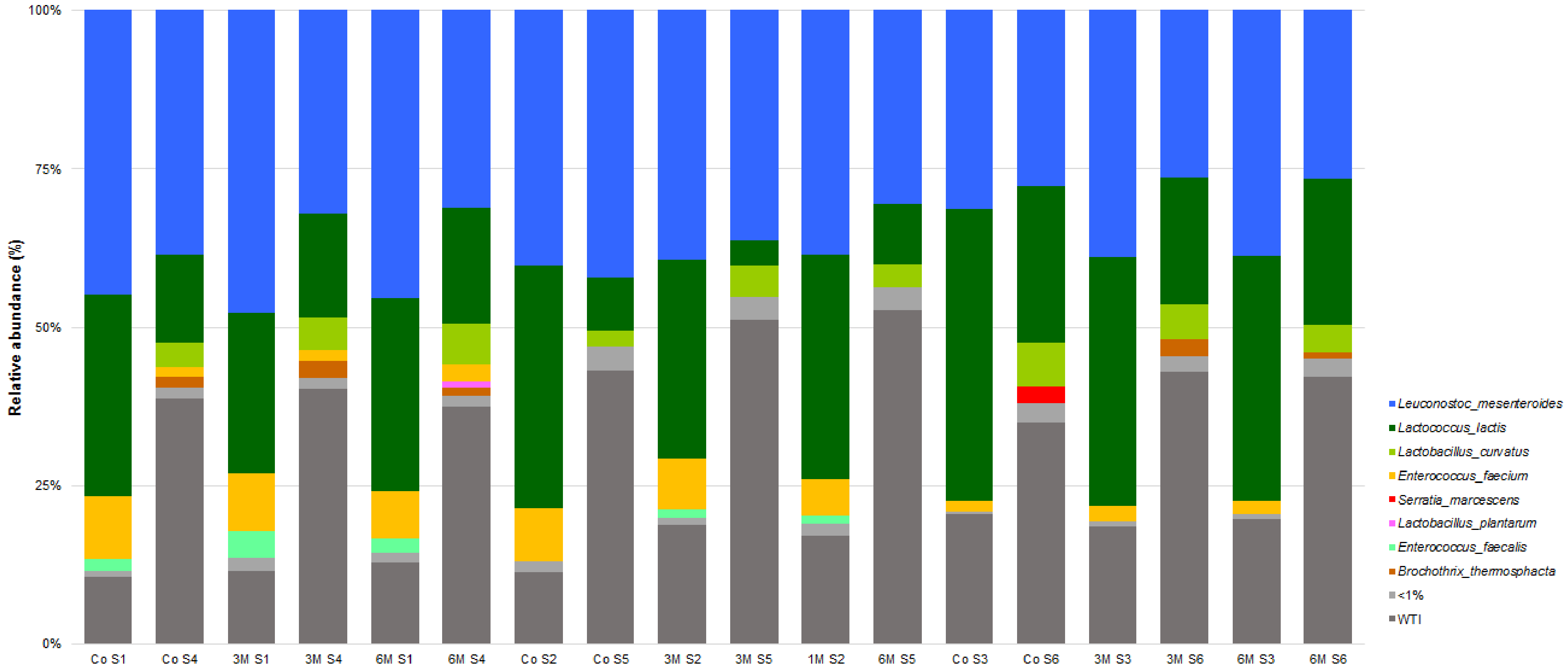

Figure S5 – Relative abundances (%) of dominant sequences (>1%) assigned to species level identified in Serra da Estrela cheese DNA samples based on partial sequence analysis of the V3−V4 region in the rRNA gene. Co refers to the commercially used cardoon by the cheese producer and 1M, 3M and 6M refers to specific cardoon genotypes. WTI refers to the percentage of sequences without taxonomic attribution to the specified taxonomic level.

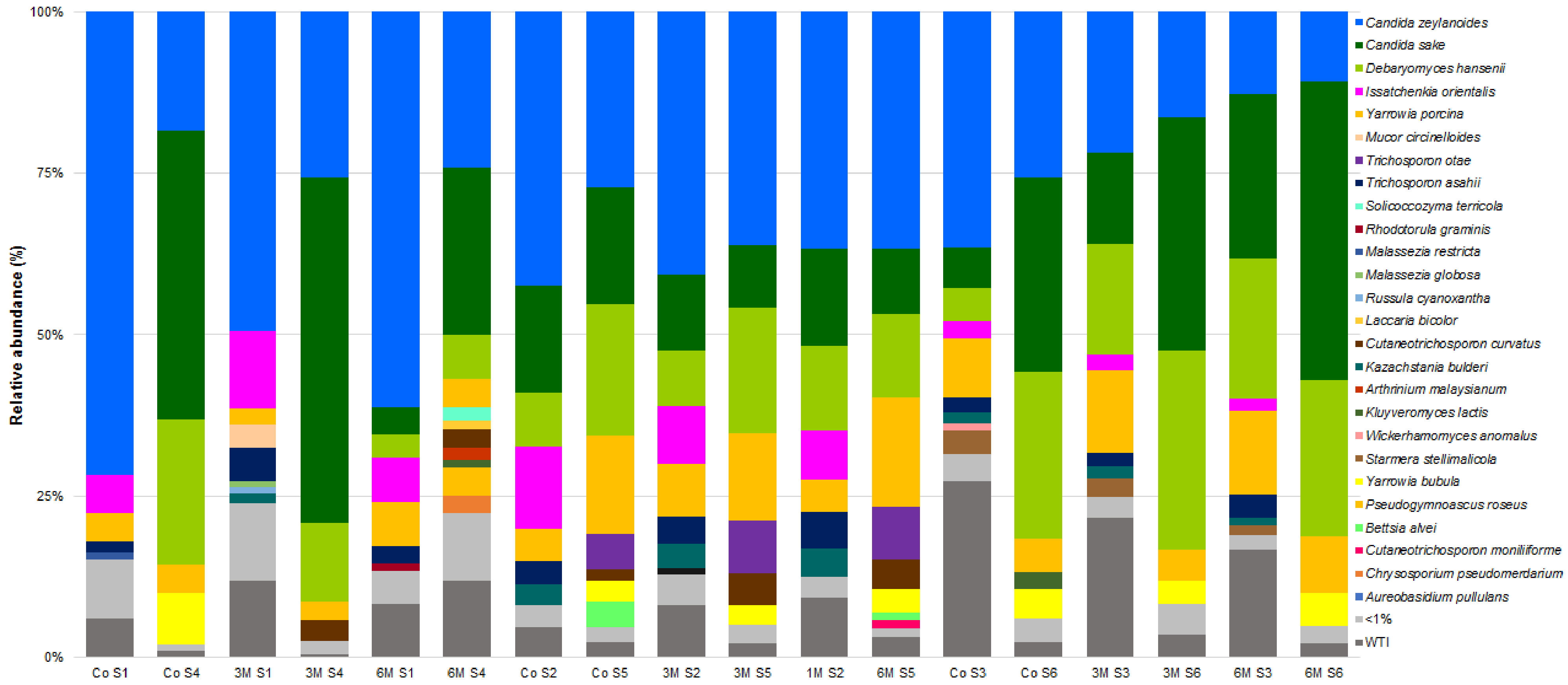

Figure S6 – Relative abundances (%) of dominant sequences (>1%) assigned to species level identified in Serra da Estrela cheese DNA samples based on partial sequence analysis of the Internal Transcribed Spacer 2 region in the rRNA gene. Co refers to the commercially used cardoon by the cheese producer and 1M, 3M and 6M refers to specific cardoon genotypes. WTI refers to the percentage of sequences without taxonomic attribution to the specified taxonomic level.

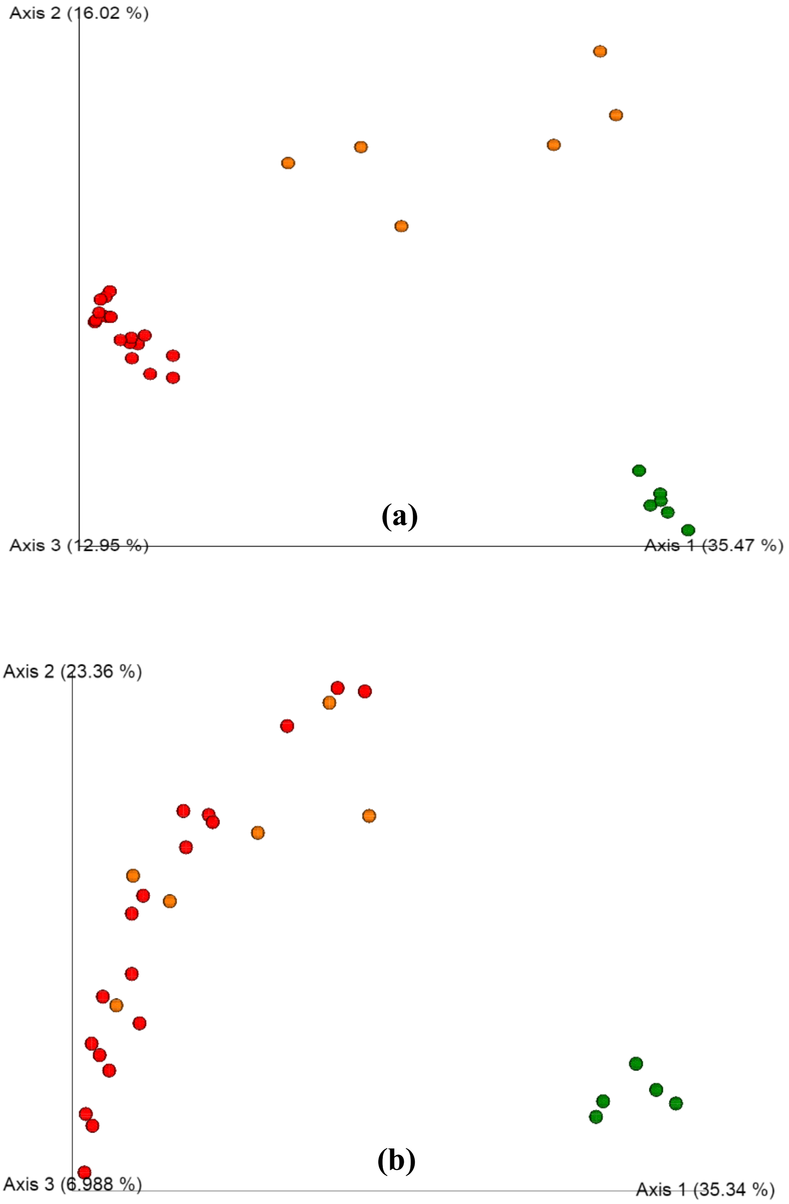

Figure S7 - Principal coordinates analysis emperor plots based on Bray-Curtis diversity metric obtained from OTU analysis of milk (orange dots), cardoon (green dots) and Serra da Estrela cheese (red dots) retrieved from DNA sequencing of (a) V3-V4 (bacterial community) and (b) Internal Transcribed Spacer 2 (fungi community) regions in the rRNA gene.

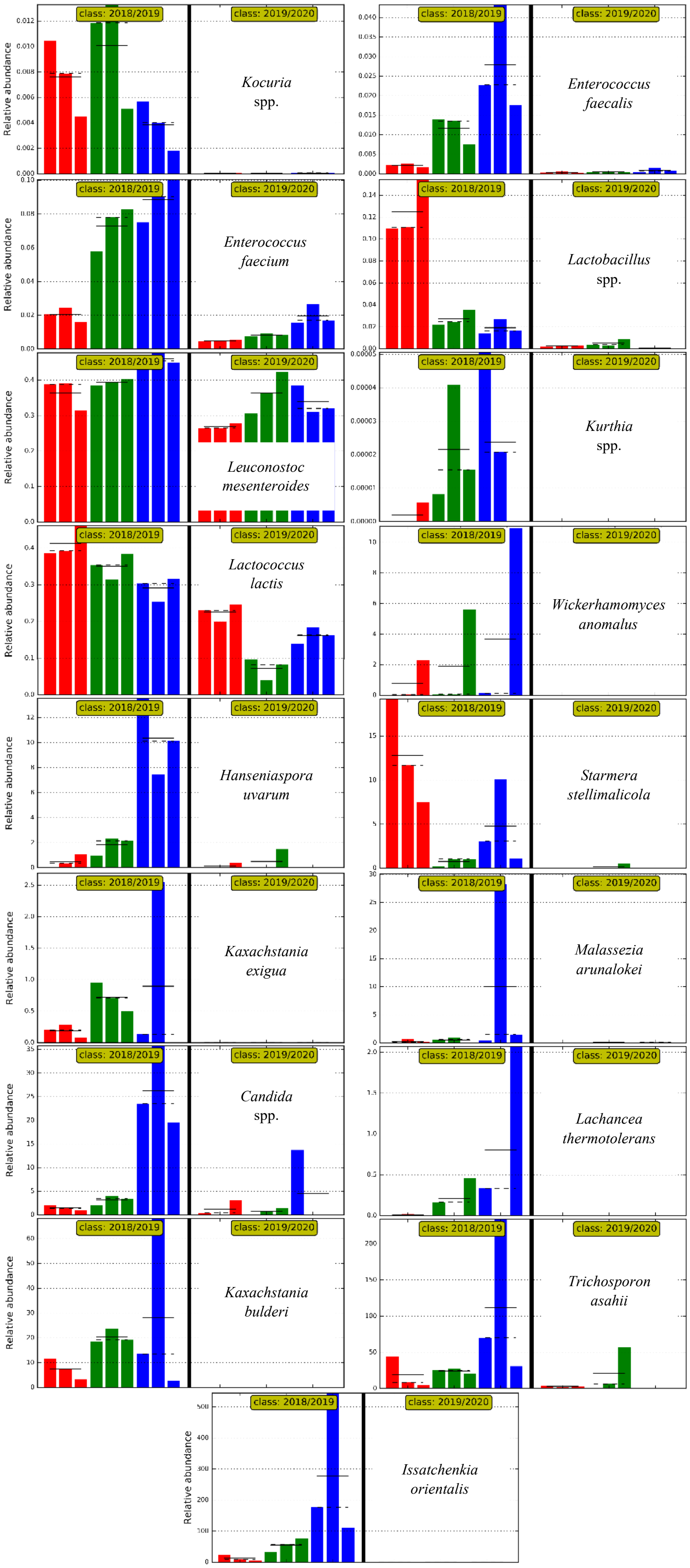

Figure S8 – Relative abundance histograms of biomarker features found for 2018/2019 Serra da Estrela cheese production campaign (class) across manufacturing periods (subclasses): autumn (blue bars), winter (green bars) and spring (red bars). Biomarker mean and median relative abundances are indicated with solid and dashed lines, respectively.

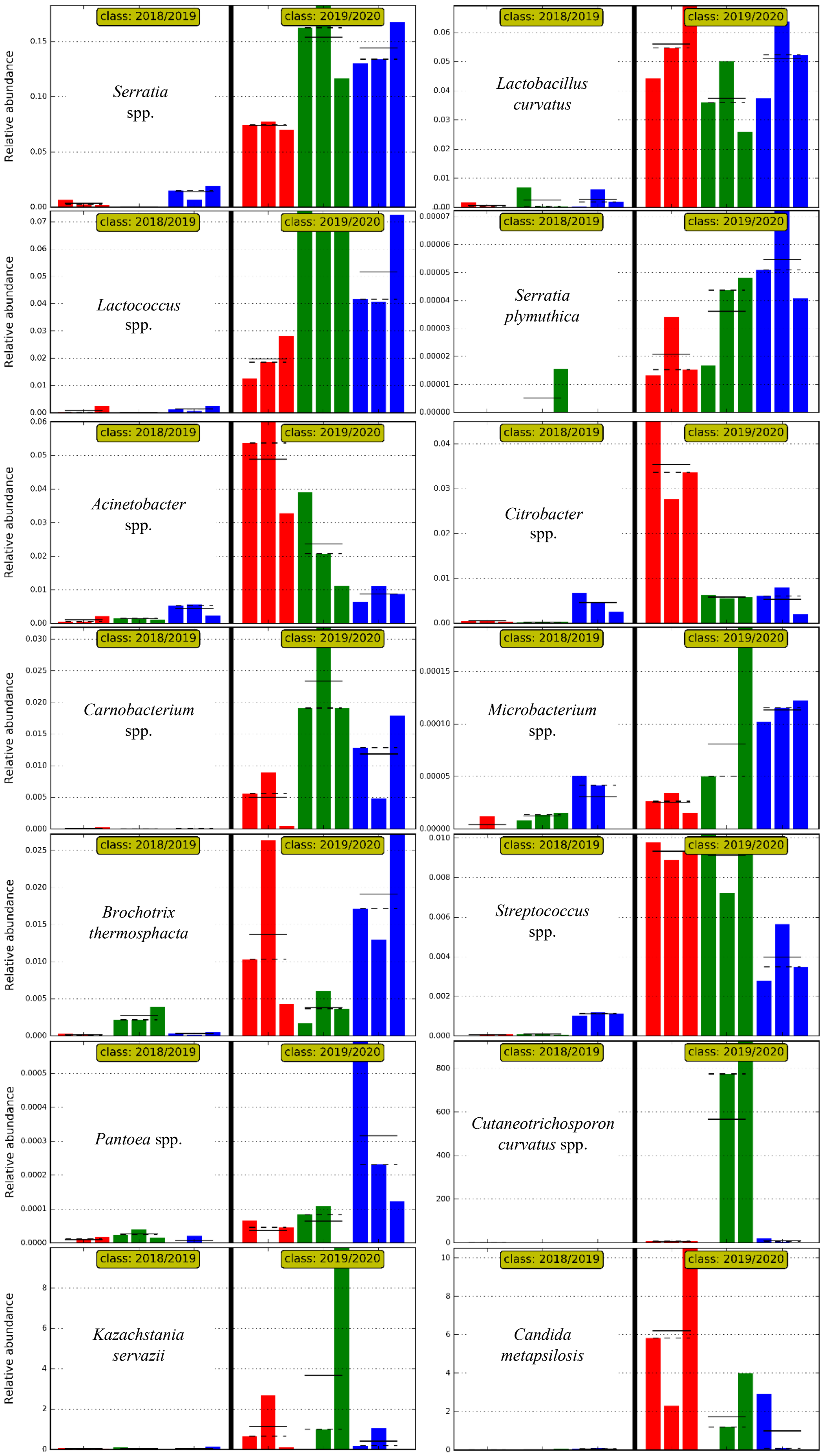

Figure S9 – Relative abundance histograms of biomarker features found for 2019/2020 Serra da Estrela cheese production campaign (class) across manufacturing periods (subclasses): autumn (blue bars), winter (green bars) and spring (red bars). Biomarker mean and median relative abundances are indicated with solid and dashed lines, respectively.
