# Supplementary material for: Serra da Estrela PDO Cheese Microbiome as Revealed by Next Generation Sequencing
Source: Microorganisms. 2021 Sep 22;9(10):2007. doi: 10.3390/microorganisms9102007 (PMC8537266; doi:10.3390/microorganisms9102007)
Supplement: Supplementary file 1 [file microorganisms-09-02007-s001.zip › Supplementary material file S2.pdf]

Table S1 – Internal Transcribed Spacer 2 sequencing summary data (raw reads, processed reads, high−quality sequences, sampling completeness, diversity indexes and richness estimators) obtained from raw ewes' milk, cardoon and cheese samples analyzed throughout this study.

Sample type	Sampling	Cardoon Genotype	Raw Reads (%)	Filtered Reads (%)	Merged Reads (%)	Dereplicated Reads (%)	Chimeric Reads (%)	Final Reads (before OTU generation) (%)	Nº of Sequences Final (%)	Nº of OTUs	Good's coverage (%)	Shannon	Simpson	Chao	ACE
Milk	S1	–	99474 (100.00%)	96053 (96.56%)	92516 (93.01%)	66927 (67.28%)	24862 (24.99%)	42065 (42.29%)	39369 (39.58%)	896	98.9	3.5927	0.6895	1607.7	1615.4
Milk	S2	–	67845 (100.00%)	65471 (96.5%)	63096 (93.0%)	50893 (75.01%)	15174 (22.37%)	35719 (52.65%)	34121 (50.29%)	496	98.9	2.3860	0.6928	1384.7	1578.5
Milk	S3	–	157400 (100.00%)	154576 (98.21%)	148819 (94.55%)	109871 (69.8%)	21078 (13.39%)	88793 (56.41%)	83891 (53.30%)	1081	99.3	3.6115	0.84	1979.6	2204.3
Milk	S4	–	46441 (100.00%)	44445 (95.7%)	42675 (91.89%)	41319 (88.97%)	1433 (3.09%)	39886 (85.89%)	38178 (82.21%)	456	99.3	2.4695	0.6934	1191.8	1234.3
Milk	S5	–	95923 (100.00%)	78930 (82.28%)	78299 (81.63%)	77510 (80.8%)	9661 (10.07%)	67849 (70.73%)	67289 (70.15%)	184	99.8	2.6874	0.7297	285.1	302.8
Milk	S6	–	95721 (100.00%)	72174 (75.4%)	70201 (73.34%)	54283 (56.71%)	1403 (1.47%)	52880 (55.24%)	52007 (54.33%)	371	99.9	3.6012	0.7647	392.7	386.1
Cardoon	S1	Commercial	115112 (100.00%)	113674 (98.75%)	107457 (93.35%)	92818 (80.63%)	5642 (4.9%)	87176 (75.73%)	80047 (69.54%)	1284	99.5	5.0758	0.9258	1415.3	1541.5
Cardoon	S1	3M	80619 (100.00%)	79859 (99.06%)	76285 (94.62%)	75207 (93.29%)	312 (0.39%)	74895 (92.9%)	70466 (87.41%)	609	99.6	3.3679	0.7636	755.0	829.0
Cardoon	S1	6M	106746 (100.00%)	105522 (98.85%)	99412 (93.13%)	90528 (84.81%)	1091 (1.02%)	89437 (83.78%)	82654 (77.43%)	1216	99.6	5.3401	0.9459	1364.2	1474.1
Cardoon	S4	Commercial	49576 (100.00%)	43966 (88.68%)	43200 (87.14%)	24563 (49.55%)	637 (1.28%)	23926 (48.26%)	23358 (47.12%)	380	99.5	4.5806	0.8866	433.3	454.4
Cardoon	S4	3M	59911 (100.00%)	52814 (88.15%)	40091 (66.92%)	29975 (50.03%)	794 (1.33%)	29181 (48.71%)	28214 (47.09%)	341	99.7	4.1841	0.8876	379.6	396.3
Cardoon	S4	6M	50812 (100.00%)	41873 (82.41%)	39390 (77.52%)	9162 (18.03%)	62 (0.12%)	9100 (17.91%)	8832 (17.38%)	324	98.3	4.2861	0.8551	518.3	557.9
Cheese	S1	Commercial	126930 (100.00%)	101447 (79.92%)	98353 (77.49%)	66306 (52.24%)	38748 (30.53%)	27558 (21.71%)	26001 (20.48%)	623	98.5	2.4795	0.4811	1405.0	1680.4
Cheese	S4	Commercial	78562 (100.00%)	76816 (97.78%)	73819 (93.96%)	64610 (82.24%)	3351 (4.27%)	61259 (77.98%)	58639 (74.64%)	519	99.3	2.2936	0.7127	1213.4	1586.7
Cheese	S1	3M	126702 (100.00%)	119178 (94.06%)	114519 (90.38%)	86262 (68.08%)	42755 (33.74%)	43507 (34.34%)	40774 (32.18%)	893	98.7	3.8518	0.7368	1681.8	2110.0

Continuation of Table S1

Cheese	S4	3M	42075 (100.00%)	33231 (78.98%)	32766 (77.88%)	31663 (75.25%)	524 (1.25%)	31139 (74.01%)	30860 (73.35%)	131	99.8	1.9548	0.632	196.6	203.7
Cheese	S1	6M	114011 (100.00%)	99311 (87.11%)	95978 (84.18%)	71998 (63.15%)	31608 (27.72%)	40390 (35.43%)	38253 (33.55%)	753	98.7	2.8492	0.612	1766.1	2049.1
Cheese	S4	6M	56836 (100.00%)	43902 (77.24%)	43111 (75.85%)	37886 (66.66%)	6726 (11.83%)	31160 (54.82%)	30639 (53.91%)	611	99.6	4.535	0.8633	663.3	679.8
Cheese	S2	Commercial	92033 (100.00%)	89970 (97.76%)	87172 (94.72%)	45402 (49.33%)	14083 (15.3%)	31319 (34.03%)	29700 (32.27%)	534	98.8	3.1157	0.7684	1504.8	1625.9
Cheese	S5	Commercial	174982 (100.00%)	140673 (80.39%)	138484 (79.14%)	125925 (71.96%)	13539 (7.74%)	112386 (64.23%)	111094 (63.49%)	332	99.8	2.9829	0.8237	447.5	493.9
Cheese	S2	3M	156789 (100.00%)	153434 (97.86%)	148883 (94.96%)	76909 (49.05%)	23869 (15.22%)	53040 (33.83%)	50430 (32.16%)	799	99.0	3.5248	0.7977	1971.2	2239.7
Cheese	S5	3M	118805 (100.00%)	101114 (85.11%)	99999 (84.17%)	94070 (79.18%)	13197 (11.11%)	80873 (68.07%)	80079 (67.40%)	254	99.8	2.8490	0.7949	438.0	376.7
Cheese	S2	6M	102310 (100.00%)	99666 (97.42%)	96711 (94.53%)	49317 (48.2%)	10918 (10.67%)	38399 (37.53%)	36503 (35.68%)	563	99.0	3.4652	0.8134	1405.5	1489.8
Cheese	S5	6M	106623 (100.00%)	92021 (86.31%)	90749 (85.11%)	87820 (82.36%)	6931 (6.5%)	80889 (75.86%)	80046 (75.07%)	210	99.9	2.9144	0.7996	285.2	282.5
Cheese	S3	Commercial	65192 (100.00%)	62815 (96.35%)	60663 (93.05%)	47342 (72.62%)	8738 (13.4%)	38604 (59.22%)	36802 (56.45%)	573	99.0	3.6242	0.8166	1438.1	1554.2
Cheese	S6	Commercial	147076 (100.00%)	125626 (85.42%)	124448 (84.61%)	109521 (74.47%)	33801 (22.98%)	75720 (51.48%)	74728 (50.81%)	315	99.8	2.7135	0.7723	445.0	421.4
Cheese	S3	3M	83443 (100.00%)	81729 (97.95%)	79135 (94.84%)	56192 (67.34%)	9138 (10.95%)	47054 (56.39%)	44908 (53.82%)	608	99.1	3.5256	0.8597	1509.5	1666.4
Cheese	S6	3M	141437 (100.00%)	114497 (80.95%)	113437 (80.2%)	106939 (75.61%)	13342 (9.43%)	93597 (66.18%)	92486 (65.39%)	388	99.8	2.7186	0.7441	555.4	482.1
Cheese	S3	6M	132217 (100.00%)	126840 (95.93%)	121719 (92.06%)	95864 (72.51%)	10617 (8.03%)	85247 (64.48%)	81032 (61.29%)	761	99.3	3.2802	0.8402	2138.0	2348.9
Cheese	S6	6M	256364 (100.00%)	221831 (86.53%)	220235 (85.91%)	196811 (76.77%)	38687 (15.09%)	158124 (61.68%)	156748 (61.14%)	369	99.9	2.4283	0.7073	507.1	525.1

Table S2 – V3−V4 sequencing summary data (raw reads, processed reads, high−quality sequences, sampling completeness, diversity indexes and richness estimators) obtained from raw ewes' milk, cardoon and cheese samples analyzed throughout this study.

Sample type	Sampling	Cardoon Genotype	Raw Reads (%)	Filtered Reads (%)	Merged Reads (%)	Dereplicated Reads (%)	Chimeric Reads (%)	Final Reads (before OTU generation) (%)	Nº of Sequences Final (%)	Nº of OTUs	Good's coverage (%)	Shannon	Simpson	Chao	ACE
Milk	S1	−	56126 (100.00%)	54293 (96.73%)	47771 (85.11%)	47771 (85.11%)	7219 (12.86%)	40552 (72.25%)	32314 (57.57%)	2316	97.2	6.5611	0.9437	2940.0	3282.6
Milk	S2	−	42538 (100.00%)	41575 (97.74%)	38579 (90.69%)	38579 (90.69%)	3074 (7.23%)	35505 (83.47%)	30771 (72.34%)	837	98.4	2.7919	0.6828	1509.6	1797.7
Milk	S3	−	45295 (100.00%)	43786 (96.67%)	39152 (86.44%)	39152 (86.44%)	2044 (4.51%)	37108 (81.93%)	32053 (70.76%)	1358	97.6	4.2295	0.8377	2277.8	2817.5
Milk	S4	−	48031 (100.00%)	47091 (98.04%)	41926 (87.29%)	41926 (87.29%)	15740 (32.77%)	26186 (54.52%)	21039 (43.80%)	1557	97.0	6.3155	0.9468	2023.2	2253.8
Milk	S5	−	108391 (100.00%)	95746 (88.33%)	86905 (80.18%)	86905 (80.18%)	24455 (22.56%)	62450 (57.62%)	57040 (52.62%)	4218	95.9	6.3964	0.9411	7300.4	8332.0
Milk	S6	−	163372 (100.00%)	158307 (96.9%)	143261 (87.69%)	143261 (87.69%)	9207 (5.64%)	134054 (82.05%)	122867 (75.21%)	9225	98.3	8.6189	0.9736	9914.7	10558
Cardoon	S1	Commercial	60066 (100.00%)	58528 (97.44%)	54518 (90.76%)	54518 (90.76%)	9118 (15.18%)	45400 (75.58%)	38017 (63.29%)	1070	99.2	5.4745	0.9354	1214.8	1329.2
Cardoon	S1	3M	52411 (100.00%)	50831 (96.99%)	46212 (88.17%)	46212 (88.17%)	542 (1.03%)	45670 (87.14%)	40018 (76.35%)	704	99.5	2.9617	0.7231	794.8	866.4
Cardoon	S1	6M	58843 (100.00%)	57688 (98.04%)	54238 (92.17%)	54238 (92.17%)	6326 (10.75%)	47912 (81.42%)	40697 (69.16%)	987	99.3	4.6453	0.8763	1078.5	1187.3
Cardoon	S4	Commercial	115878 (100.00%)	109107 (94.16%)	105337 (90.9%)	105337 (90.9%)	37368 (32.25%)	67969 (58.66%)	62720 (54.13%)	2443	99.0	6.9580	0.9641	2804.0	2984.0
Cardoon	S4	3M	147117 (100.00%)	141540 (96.21%)	135114 (91.84%)	135114 (91.84%)	14319 (9.73%)	120795 (82.11%)	113739 (77.31%)	2510	99.4	4.8836	0.8747	2858.8	3076.4
Cardoon	S4	6M	97563 (100.00%)	92748 (95.06%)	90645 (92.91%)	90645 (92.91%)	4605 (4.72%)	86040 (88.19%)	81210 (83.24%)	2450	99.1	5.4763	0.8933	2881.8	3118.2
Cheese	S1	Commercial	48177 (100.00%)	47228 (98.03%)	44621 (92.62%)	44621 (92.62%)	4679 (9.71%)	39942 (82.91%)	35980 (74.68%)	719	98.6	3.1106	0.7392	2152.9	2488.0
Cheese	S4	Commercial	83977 (100.00%)	82980 (98.81%)	77469 (92.25%)	77469 (92.25%)	10416 (12.4%)	67053 (79.85%)	58832 (70.06%)	972	99.0	4.2863	0.8418	1782.3	2156.4

Continuation of Table S2.

Cheese	S1	3M	68875 (100.00%)	67732 (98.34%)	63965 (92.87%)	63965 (92.87%)	10344 (15.02%)	53621 (77.85%)	48108 (69.85%)	950	98.6	3.2143	0.7354	2575.0	3288.3
Cheese	S4	3M	104805 (100.00%)	97511 (93.04%)	95331 (90.96%)	95331 (90.96%)	19075 (18.2%)	76256 (72.76%)	73388 (70.02%)	687	99.5	4.1086	0.8628	1314.6	1362.4
Cheese	S1	6M	110948 (100.00%)	108226 (97.55%)	102731 (92.59%)	102731 (92.59%)	15420 (13.9%)	87311 (78.7%)	79159 (71.35%)	1381	98.9	3.2394	0.7414	3011.9	3654.5
Cheese	S4	6M	98029 (100.00%)	90865 (92.69%)	88942 (90.73%)	88942 (90.73%)	17421 (17.77%)	71521 (72.96%)	69254 (70.65%)	665	99.5	4.3571	0.8705	1287.7	1360.4
Cheese	S2	Commercial	88993 (100.00%)	88084 (98.98%)	83329 (93.64%)	83329 (93.64%)	12001 (13.49%)	71328 (80.15%)	64569 (72.56%)	1129	98.8	3.0355	0.7487	2930.5	3609.2
Cheese	S5	Commercial	37749 (100.00%)	35483 (94.0%)	34612 (91.69%)	34612 (91.69%)	12651 (33.51%)	21961 (58.18%)	20798 (55.10%)	351	99.1	4.0571	0.8161	646.0	732.5
Cheese	S2	3M	103169 (100.00%)	102248 (99.11%)	96661 (93.69%)	96661 (93.69%)	15672 (15.19%)	80989 (78.5%)	73517 (71.26%)	1280	98.8	3.2758	0.7781	3234.3	3769.3
Cheese	S5	3M	82512 (100.00%)	76630 (92.87%)	74920 (90.8%)	74920 (90.8%)	26955 (32.67%)	47965 (58.13%)	45757 (55.45%)	634	99.2	4.192	0.8465	1360.0	1513.0
Cheese	S2	6M	165427 (100.00%)	163972 (99.12%)	155553 (94.03%)	155553 (94.03%)	20895 (12.63%)	134658 (81.4%)	122303 (73.93%)	1628	99.1	3.2529	0.7753	3558.1	4110.1
Cheese	S5	6M	116626 (100.00%)	103155 (88.45%)	100486 (86.16%)	100486 (86.16%)	37187 (31.89%)	63299 (54.28%)	59725 (51.21%)	740	99.4	4.4770	0.8801	1322.4	1416.2
Cheese	S3	Commercial	235051 (100.00%)	232747 (99.02%)	220338 (93.74%)	220338 (93.74%)	30823 (13.11%)	189515 (80.63%)	174208 (74.11%)	1954	99.4	3.0052	0.7646	3390.9	3966.2
Cheese	S6	Commercial	92302 (100.00%)	84894 (91.97%)	82744 (89.64%)	82744 (89.64%)	14930 (16.18%)	67814 (73.47%)	65363 (70.81%)	512	99.6	4.095	0.8669	950.4	1034.3
Cheese	S3	3M	108770 (100.00%)	107671 (98.99%)	102563 (94.29%)	102563 (94.29%)	13778 (12.67%)	88785 (81.63%)	81530 (74.96%)	1234	99.0	3.0551	0.7539	2962.8	3771.9
Cheese	S6	3M	84806 (100.00%)	80347 (94.74%)	78226 (92.24%)	78226 (92.24%)	17240 (20.33%)	60986 (71.91%)	58660 (69.17%)	615	99.5	4.4313	0.8883	1163.6	1222.1
Cheese	S3	6M	75411 (100.00%)	74717 (99.08%)	71003 (94.15%)	71003 (94.15%)	10760 (14.27%)	60243 (79.89%)	55467 (73.55%)	904	98.9	3.1189	0.7595	2560.4	3106.2
Cheese	S6	6M	105743 (100.00%)	101656 (96.13%)	98744 (93.38%)	98744 (93.38%)	20369 (19.26%)	78375 (74.12%)	75630 (71.52%)	683	99.6	4.3576	0.8809	1083.7	1193.8
